# Magnesium Levels Modify the Effect of Lipid Parameters on Carotid Intima Media Thickness

**DOI:** 10.3390/nu12092631

**Published:** 2020-08-28

**Authors:** Serafi Cambray, Merce Ibarz, Marcelino Bermudez-Lopez, Manuel Marti-Antonio, Milica Bozic, Elvira Fernandez, Jose M. Valdivielso

**Affiliations:** 1 Vascular and Renal Translational Research Group, Institute for Biomedical Research Pifarré Foundation, IRBLleida Av. Rovira Roure 80, 25198 Lleida, Spain; mbermudez@irblleida.cat (M.B.-L.); mmartia@irblleida.cat (M.M.-A.); milica.bozic@irblleida.udl.cat (M.B.); elvirafgiraldez@gmail.com (E.F.); 2 Indicators and Specifications of the Quality in the Clinical Laboratory Group, Institute for Biomedical Research Pifarré Foundation, IRBLleida, 25198 Lleida, Spain; mibarz.lleida.ics@gencat.cat

**Keywords:** magnesium, cholesterol, atherosclerosis, first-order interaction, cardiovascular risk

## Abstract

Classical risk factors of atherosclerosis in the general population show paradoxical effects in chronic kidney disease (CKD) patients. Thus, low low-density lipoprotein (LDL) cholesterol levels have been associated with worse cardiovascular outcomes. Magnesium (Mg) is a divalent cation whose homeostasis is altered in CKD. Furthermore, Mg levels have been associated with cardiovascular health. The present study aims to understand the relationships of Mg and lipid parameters with atherosclerosis in CKD. In this analysis, 1754 participants from the Observatorio Nacional de Atherosclerosis en Nefrologia (NEFRONA) cohort were included. Carotid intima media thickness (cIMT) was determined in six arterial territories, and associated factors were investigated by linear regression. cIMT correlated positively with being male, Caucasian, a smoker, diabetic, hypertensive, dyslipidemic and with increased age, BMI, and triglyceride levels, and negatively with levels of HDL cholesterol. First-order interactions in linear regression analysis showed that Mg was an effect modifier on the influence of lipidic parameters. Thus, cIMT predicted values were higher when triglycerides or LDL levels were high and Mg levels were low. On the contrary, when Mg levels were high, this effect disappeared. In conclusion, Mg acts as an effect modifier between lipidic parameters and atherosclerotic cardiovascular disease. Therefore, Mg levels, together with lipidic parameters, should be taken into account when assessing atherosclerotic risk.

## 1. Introduction

According to the Cardiovascular Disease Statistics from the European Society of Cardiology, in 2015 there were 11 million new cases of cardiovascular disease (CVD) reported in Europe, and the majority of countries showed an increase in cases from 1990 [1]. Its prevalence was reported to be 83.5 million people, of which 35.7 million people showed peripheral vascular disease [1]. Moreover, CVD also led to 64 million disability-adjusted life years in the European population [1]. 

Among risk factors associated with CVD, plasma lipids play a key role in the initiation and progression of atheromatous disease [2]. Epidemiological studies show that increased concentrations of low-density lipoprotein cholesterol (LDL-C) are associated with an increased risk of cardiovascular events [3,4], while the contrary effect has been demonstrated for high-density lipoprotein cholesterol [5] (HDL-C). Triglyceride levels are inversely associated with HDL-C levels [6] and, despite not showing atherogenic properties per se, are considered to be an important biomarker of CVD, due to their association with atherogenic remnant lipoproteins containing apo CIII [7]. Triglycerides have important implications for chronic kidney disease (CKD) patients, a population that presents a high incidence of atherosclerotic events [8]. Additionally, traditional lipid profiles are not associated with increased cardiovascular risk in this population. CKD patients present low levels of HDL-C and hypertriglyceridemia [9], but levels of LDL-C or total cholesterol are not usually modified, or are even low in advanced stages [10]. Some works point to the importance of LDL-C particle size [11] or the levels of oxidized LDL-C [12]. Nevertheless, the link between altered lipid metabolism and the higher presence of atheromasias in CKD patients is not clear. 

Currently, to prevent cardiovascular events in high risk populations, most efforts have been directed towards lowering plasma LDL-C and triglycerides, by means of pharmacological treatment [13], dietary intervention [14,15], or diet supplements [16,17,18]. Among diet supplements, magnesium (Mg) was one of the first to be used for lowering serum lipids. During the 1980s, it was demonstrated that Mg deficiency was associated with hypertension and vascular calcifications, while increasing its dietary intake prevented atheroma in experimental animals with normal renal function [19], and in experimental uremia [20]. Later on, during the 1990s, different studies in patients showed that lower Mg plasma concentrations were associated with atherosclerosis [21], and that Mg supplementation lowered plasma cholesterol, LDL, and triglyceride concentrations [22,23]. Recent studies also found an inverse correlation between Mg levels, carotid intima–media thickness (cIMT) [24,25] and peripheral artery disease [26,27], while many epidemiological studies and clinical trials reported Mg as a key player in cardiovascular health [28,29,30,31], even in hemodialysis patients [32]. However, randomized clinical trials with Mg supplementation have yielded conflicting results [33,34]. CKD patients often present an altered Mg balance. Thus, the decrease in glomerular filtration rate (GFR) can induce Mg retention, whereas in other cases tubular dysfunction and diuretic use can induce hypomagnesemia. Therefore, the altered Mg levels, alongside the specific dyslipidemia found in those patients, make them a very interesting group to investigate the possible interactions of both variables in atherosclerosis.

Despite the fact that some data showed that Mg levels can affect cIMT and that, according to some studies, it could also affect plasma lipid concentrations, no studies have analyzed the possible interactions of both variables in atherosclerosis. In the present study, we aim to study the impact of Mg on cIMT, and how Mg interacts with plasma lipids and other known CVD risk factors in a CKD population form the Observatorio Nacional de Atherosclerosis en Nefrologia (NEFRONA) cohort. 

## 2. Materials and Methods 

### 2.1. Study Design

The NEFRONA study was designed to assess the utility of noninvasive vascular imaging techniques and plasma biomarkers to predict cardiovascular events and mortality in CKD patients [35,36]. Briefly, CKD and non-CKD volunteers aged 18 to 75 years were recruited throughout Spanish primary care centers and renal units from 2009 to 2012. Patients with a history of CVD, remarkable carotid stenosis, active infections (tuberculosis and human immunodeficiency virus), pregnancy, less than twelve month of life expectancy, and with any organ transplantation or carotid artery surgery were excluded from the study. Of the NEFRONA study, 1754 subjects had available serum samples to measure Mg levels. Out of those, 40 presented with missing data on CKD status or cIMT values. Thus, 1754 volunteers were used in the present study. In total, 1542 presented with CKD (629 Stage 3; 528 Stages 4–5; 385 dialysis), and 212 were non-CKD controls (glomerular filtration rate > 60 mL/min/1.73 m^2^). The ethics committee of each hospital approved the protocol of the study, and all volunteers were included after signing an informed consent. The research followed the principles of the Declaration of Helsinki. Non-CKD controls were included as per the protocol of the NEFRONA study and as a reference with normal Mg and lower cIMT values. 

### 2.2. Clinical Data and Mg Determination

A nurse and two specifically trained technicians collected the following data: gender, age, body mass index, systolic and diastolic blood pressure (SBP and DBP), pulse pressure, and smoking status. Information about presence of diabetes, hypertension, and dyslipidemia was obtained from clinical records. Fasting blood samples were also collected by the same team and were stored at −80 °C in the Biobank of the RedInRen in the University of Alcala de Henares (Madrid). Biochemical analysis was performed as described previously [37]. The determination of Mg in serum samples was performed using the Mg reagent from Beckman Coulter (Brea, CA USA; Ref. OSR6189), following the manufacturer’s instructions. 

### 2.3. Atherosclerosis Assessment

Atherosclerosis assessment was performed as previously described [38]. Briefly, carotid ultrasound measurements were performed in three territories of both carotid arteries (bifurcation, internal, and common carotid arteries). Plaques were defined according to the Mannheim Carotid Intima–Media Thickness (cIMT) Consensus and the American Society of Echocardiography as a cIMT lumen protrusion ≥ 1.5 mm [39,40]. More extended protocols and data about plaque prevalence in the entire NEFRONA cohort have been published previously [37]. In the present analysis, we used the average cIMT of the territories that did not show atheroma plaque.

### 2.4. Statistical Analysis

Absolute frequencies (and percentage) or mean (and standard deviation) were used to describe qualitative and quantitative variables, respectively. Pearson correlation coefficients were calculated to analyze the relationships between cIMT and Mg levels with other clinical variables. Multivariate regression linear models were used to assess the association of clinical variables with cIMT. To specifically assess the joint association of Mg with the rest of the explanatory variables on cIMT, all possible first-order interactions were considered in the model, and a backwards stepwise algorithm was used to select the significant ones. A graph showing the predicted values of cIMT when the rest of the variables of the model are set to zero, and showing the interactions of continuous values of lipid parameters and Mg levels was drawn. All analyses were performed using R, setting the threshold of significance at 0.05. 

## 3. Results

### 3.1. Clinical Characteristics

The study comprised 1754 volunteers with a mean age of 58.6 ± 12.7 years. Significant differences were found between CKD stages in all the parameters of the study, except in the percentage of smokers, which was not significantly different between CKD stages. Interestingly, Mg levels increased as renal function decreased, whereas cIMT levels showed the opposite tendency. LDL levels also showed a significant tendency to decrease as renal function impairment worsened (Table 1).

### 3.2. Correlations of Clinical Variables with cIMT and Mg Levels

Results for the correlation matrix of cIMT and Mg levels with the clinical variables of the study are shown in Table 2. cIMT correlated positively with being male, Caucasian, smoker, diabetic, hypertensive, dyslipidemic and with increased age, BMI, and triglyceride levels. Furthermore, it correlated negatively with levels of HDL cholesterol. Interestingly, no significant correlation was found with total or LDL cholesterol levels. Mg levels positively correlated with the presence of hypertension, and negatively with being Caucasian, diabetic, and with level of BMI. The correlation matrix of the variables with cIMT was calculated for the controls (Supplementary Table S1) as a sensitivity analysis. Some of the significant correlations were lost (due to the lower sample size), but most of the coefficients were similar.

### 3.3. Association of Clinical Variables with cIMT

The results from the multivariate main effects model, which included all the variables studied, showed that age, being male or a smoker, pulse pressure, potassium, and CKD stage were factors that were significantly associated with cIMT. Mg levels did not reach statistical significance in this model, underlying the fact that Mg had no association with cIMT when considering its effect in the multivariate model independently of the rest of variables (Table 3). However, interestingly, the results from the model including the interactions revealed that Mg levels were significantly associated with cIMT, when interactions with cholesterol levels (total, LDL-C, and HDL-C) and triglycerides are taken into account. Sex, age, tobacco, pulse pressure, CKD stage, and potassium levels were significantly associated with cIMT in this multivariate model (Table 4).

### 3.4. Visualization of the Association of the Interaction of Mg and Plasma Lipids with cIMT

**Figure 1:**
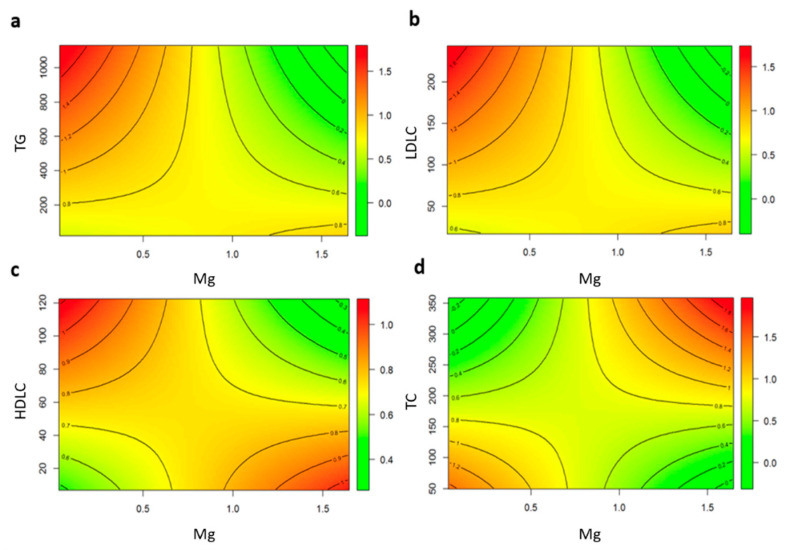
Interaction Graphs for Continuous Data. (**a**) Predicted cIMT with different levels of triglycerides (TG) and magnesium (Mg). (**b**) Predicted cIMT with different levels of LDL cholesterol (LDLC) and magnesium. (**c**) Predicted cIMT with different levels of HDL cholesterol (HDLC) and magnesium. (**d**) Predicted cIMT with different levels of total cholesterol (TC) and magnesium.

To better visualize the interactions between plasma lipids and Mg, we performed an interaction graph for continuous data. As seen in Figure 1a, cIMT predicted values were higher when triglycerides levels were high and Mg levels were low. On the contrary, when Mg levels were higher than 1 mmol/L, the relationship between high triglyceride levels and increased cIMT disappeared. A similar tendency was seen when LDL-C levels, Mg, and cIMT were plotted (Figure 1b). The interaction between HDL-C and Mg showed that the protective effect of high HDL-C levels disappears when Mg levels are low (Figure 1c). Finally, a paradoxical effect of total cholesterol levels is depicted, predicting higher cIMT when both total cholesterol and Mg are high (Figure 1d). 

## 4. Discussion

The present study identifies, for the first time, the role of Mg as an effect modifier on the association of lipid parameters with cIMT in CKD patients. Thus, high LDL and triglyceride levels affect cIMT only when Mg levels are low. In the same model, high HDL levels associate with lower cIMT only when Mg levels are high. This result could be behind some of the paradoxical effects of lipid parameters on atherosclerosis in renal patients, in which Mg levels are often modified by the disease.

Hypertriglyceridemia and high cholesterol levels are related to atherosclerosis [2,41], and current mathematical models estimate that lipid-lowering therapies could avoid a substantial number of cardiovascular events [42]. Among the different dietary supplements proposed for lipid-lowering treatment, Mg has been used not only to prevent cardiovascular events in patients suffering from chronic kidney disease [43], but also for metabolic syndrome treatment [44] as well as to improve cardiovascular health in overweight and obese people [45]. In renal patients, however, the relationship between lipid parameters and atherosclerosis is not so clear. Thus, high LDL cholesterol levels have sometimes been found to be unrelated to cardiovascular disease in CKD patients. In other cases, the phenomenon called reverse causality has also been shown in this population, finding an association of lower LDL levels with higher cardiovascular risk [46]. Although malnutrition has been shown to partially explain this paradoxical effect, hypomagnesemia, which is associated with malnutrition, could be part of the exact mechanism [47]. The same different effect on cardiovascular risk has also been observed with HDL, which seems to lose its association with cardiovascular risk as renal function declines [48]. This report also shows that the interaction between Mg and triglycerides has an impact on cIMT. Pioneering work from the ARIC study found a relationship between serum Mg levels and cIMT that was maintained after adjusting for age, but that disappeared after adjusting for other risk factors [49]. Indeed, and in agreement with previous literature reports, Mg has been recently identified as a possible independent risk factor for carotid atherosclerosis [50], and a meta-analysis showed an inverse relationship between circulating and dietary Mg and cardiovascular risk [30]. As far as we know, none of the works that identified a relationship between serum Mg levels and cIMT have considered the impact of Mg–lipid interaction on cIMT.

One of the possible explanations for our results is that Mg is an important antioxidant, and its deficiency has been related to an increase in oxidative stress biomarkers [51], and to an increase in lipid peroxidation [52,53]. High levels of these molecules have been associated with cardiovascular events [54]. A proposed mechanism of its atherogenic potential includes the recruitment and retention of macrophages, the secretion of cytokines by macrophages and endothelial cells, the proliferation of smooth muscle cells, and lymphocyte chemotaxis [55]. Therefore, according to our findings, high Mg levels could mitigate lipid peroxidation even with high plasma lipid profiles, thereby reducing its impact on cIMT. Another possible mechanism to explain our results could be the prevention of the oxidation of LDL by Mg. There is a clear relationship between the levels of oxidized LDL and atheromatous disease [56], and CKD patients showed increased levels of oxidized LDL [57]. In addition, it has been reported that oxidized LDL concentrations are higher in subjects with low Mg levels [58,59]. Thus, future studies should aim to correlate Mg levels with oxidized LDL levels and atheromatous disease in CKD patients. Finally, it is worth mentioning that many studies have proposed an active role of Mg in retarding vascular calcification of the medial arterial layer. However, this process is not related to lipid peroxidation, but to the binding of phosphate, decreasing calcium phosphate deposition [60], and regulating smooth muscle cells transdifferentiation [61].

This study has some limitations. First, its cross-sectional nature impeded us from making predictions—only associations. Furthermore, and in order to obtain a relatively wide degree of variation in Mg levels, a population with controls, different stages of CKD, and hemodialysis were included. This last group is especially sensible for the current analysis due to its susceptibility to present atherosclerotic disease and a plethora of additional pro-atherogenic factors (such as dialysis vintage, type of dialysis, etc.) that cannot be included in the whole analysis as possible confounders. However, CKD stage was included in the logistic model and did not reach statistical significance, allowing for the extrapolation of our results to our whole population. The second limitation is that we only measured plasma Mg, whose levels could differ from intracellular Mg [62,63] (which is responsible for the majority of reactions involving it as coenzyme [31]), and from levels obtained by the twenty-four hour excretion of magnesium in urine, which could more accurately reflect Mg balance. However, on the one hand, plasma lipid peroxidation is the possible mechanism by which Mg could be involved in the modification of cardiovascular events [54], and therefore serum Mg levels could better correlate with cIMT than tissue levels. On the other hand, Mg excretion in urine should be carefully taken in CKD patients [64]; consequently, for the current study, plasma Mg is a valid quantification method. As for the strengths of the current work, we would like to hallmark the relatively large cohort, which allowed us to adjust the regression models by ten different variables.

In summary, the current study shows an interaction of Mg with lipid parameters that modifies its effect on cITM. This result could shed some light on the effect of lipid levels on atherosclerosis in conditions in which Mg levels are modified and highlight that the important effect of this mineral on cardiovascular physiology could be more complex than initially thought. Although this analysis suggests around 1 nmol/L as the cutoff point of serum Mg that could influence the deleterious effect of lipids on atherosclerosis, further specifically designed studies are needed. In conclusion, Mg levels should be measured together with lipid parameters in order to assess the risk of atherosclerosis, especially in situations in which alterations in serum Mg are expected.

## Figures and Tables

**Table 1:** Clinical characteristics of the cohort.

Variable	All *n* = 1754 (100%)	Control *n* = 212 (12.1%)	CKD 2–3 *n* = 629 (35.9%)	CKD 4–5 *n* = 528 (30.1%)	Dialysis *n* = 385 (21.9%)	*p*-Value (CKD Groups)	*p*-Value (Trend CKD Groups)
Sex (Male)	1040 (59.3%)	100 (47.2%)	425 (67.6%)	299 (56.6%)	216 (56.1%)	<0.001	0.512
Race (Caucasian)	1704 (97.1%)	209 (98.6%)	620 (98.6%)	514 (97.3%)	361 (93.8%)	<0.001	<0.001
Age, years	58.6 (12.7)	51.7 (12.4)	62.15 (11.2)	58.8 (12.5)	56.4 (13.6)	<0.001	0.001
Smoker (Yes)	977 (55.7%)	123 (58%)	363 (57.7%)	279 (52.8%)	212 (55.1%)	0.347	0.211
Diabetes (Yes)	373 (21.3%)	0 (0%)	170 (27%)	127 (24.1%)	76 (19.7%)	<0.001	0.003
Hypertension (Yes)	1498 (85.4%)	67 (31.6)	569 (90.5%)	507 (96%)	355 (92.8%)	<0.001	<0.001
Dyslipidemia (Yes)	1170 (66.7%)	52 (24.5)	467 (74.2%)	399 (75.6%)	252 (65.5%)	<0.001	<0.001
BMI (kg/m^2^)	28.3 (5.21)	27.7 (4.5)	29.2 (4.7)	28.5 (5.5)	26.9 (5.6)	<0.001	0.026
SBP (mmHg)	138 (20.4)	131 (17.9)	138 (18.7)	141 (18.9)	138 (24.8)	0.004	<0.001
DBP (mmHg)	79.8 (10.9)	79 (10.2)	79.6 (9.7)	81 (10.2)	79 (13.5)	<0.001	0.792
Pulse pressure (mmHg)	58.7 (16.7)	52.1 (12.6)	58.8 (15.4)	60.3 (16.8)	59.9 (19.4)	<0.001	<0.001
Total Cholesterol (mg/dL)	180 (39.9)	203 (32.3)	184 (37.5)	177 (38.5)	164 (42.2)	<0.001	<0.001
HDL Cholesterol (mg/dL)	50.1 (14.9)	52.9 (13.8)	50.8 (14.8)	50.1 (15.1)	46.9 (15.1)	<0.001	<0.001
LDL Cholesterol (mg/dL)	104 (34.0)	127 (30.2)	107 (31.6)	100 (33.0)	91.9 (34.5)	<0.001	<0.001
Triglycerides (mg/dL)	141 (84.6)	116 (71.7)	145 (83.5)	143 (88.6)	146 (85.5)	<0.001	<0.001
Glucose (mg/dL)	107 (42.1)	96 (12.4)	110 (38.4)	107 (45.1)	108 (52.5)	<0.001	0.003
Calcium (mmol/L)	2.33 (0.152)	2.34 (0.094)	2.37 (0.119)	2.34 (0.144)	2.24 (0.197)	<0.001	<0.001
Phosphorus (mmol/L)	1.259 (0.332)	1.09 (0.158)	1.06 (0.18)	1.292 (0.245)	1.55 (0.442)	<0.001	<0.001
Sodium (mmol/L)	141 (2.93)	141 (2.25)	141 (2.65)	141 (2.80)	139 (3,22)	<0.001	<0.001
Potassium (mmol/L)	4.79 (0.60)	4.4 (0.39)	4.7 (0.50)	4.9 (0.54)	4.9 (0.80)	<0.001	<0.001
Magnesium (mmol/L)	0.83 (0.15)	0.82 (0.08)	0.80 (0.12)	0.84 (0.14)	0.88 (0.22)	<0.001	<0.001
cIMT	0.73 (0.14)	0.70 (0.13)	0.75 (0.14)	0.71 (0.14)	0.72 (0.14)	<0.001	0.569

Absolute frequency (percentage) and mean (standard deviation) are shown for qualitative and quantitative variables, respectively. Abbreviations: Body Mass Index (BMI), Systolic Blood Pressure (SBP), Diastolic Blood Pressure (DBP), Carotid Intima–Media Thickness (cIMT), Chronic Kidney Disease (CKD). LDL—low-density lipoprotein; HDL—high density lipoprotein.

**Table 2:** Bivariate correlation coefficients between cIMT and Magnesium levels with other variables.

	cIMT		Magnesium	
Variable	r	*p-*Value	r	*p-*Value
Sex (Male)	0.223	<0.001	−0.003	0.906
Race (Caucasian)	0.083	0.001	−0.079	0.001
Age, years	0.528	<0.001	−0.035	0.141
Smoker	0.105	<0.001	−0.018	0.445
Diabetes	0.157	<0.001	−0.070	0.003
Hypertension	0.137	<0.001	0.053	0.028
Dyslipidemia	0.094	<0.001	0.015	0.536
BMI	0.158	<0.001	−0.053	0.027
Total Cholesterol	0.021	0.392	−0.043	0.075
HDL Cholesterol	−0.071	0.007	0.001	0.967
LDL Cholesterol	0.007	0.781	−0.009	0.724
Triglycerides	0.082	0.001	0.009	0.709
Magnesium	−0.003	0.914	-	-
cIMT	-	-	−0.003	0.914

Pearson’s correlation coefficients (r) and *p*-value are shown for each variable. Abbreviations: Body Mass Index (BMI), Carotid Intima–Media Thickness (cIMT).

**Table 3:** Multivariate linear main effects model for cIMT.

	All Cohort		
	Beta	SE	*p*-Value
Intercept	0.14	0.18	0.43
Magnesium	0.002	0.02	0.92
Sex, male	0.034	0.007	<0.00001
Race, Caucasian	0.006	0.02	0.74
Age	0.005	0.0003	<0.00001
Current smoker	0.02	0.007	0.01
Diabetes	−0.0002	0.01	0.98
Hypertension	0.01	0.01	0.35
Dyslipidemia	−0.005	0.007	0.49
BMI	0.0003	0.0007	0.65
Pulse pressure	0.0007	0.0002	0.0004
CKD Stage, 2–3	−0.03	0.013	0.015
CKD Stage, 4–5	−0.06	0.014	0.00006
CKD Stage, dialysis	−0.016	0.015	0.31
Total Cholesterol	0.0005	0.0004	0.25
HDL Cholesterol	−0.0006	0.0005	0.24
LDL Cholesterol	−0.0002	0.0004	0.57
Triglycerides	−0.00002	0.00009	0.82
Glucose	0.0001	0.0001	0.25
Calcium	−0.001	0.006	0.82
Phosphorus	−0.002	0.004	0.59
Sodium	0.0007	0.001	0.53
Potassium	0.014	0.006	0.016

Estimated parameters (beta), standard error (SE) and *p*-value shown for each variable. Abbreviations: Body Mass Index (BMI), Chronic Kidney Disease (CKD).

**Table 4:** Multivariate linear effects model for cIMT with first-order interactions.

	All Cohort		
	Beta	SE	*p*-Value
Intercept	0.35	0.089	0.00007
Sex, male	0.04	0.007	<0.00001
Age (years)	0.006	0.0003	<0.00001
Current smoker	0.018	0.007	0.006
Pulse pressure	0.0007	0.0002	0.0002
CKD stage, 2–3	−0.027	0.01	0.01
CKD stage, 4–5	−0.05	0.01	0.00001
CKD stage, dialysis	−0.014	0.01	0.26
Magnesium	−0.12	0.099	0.23
Total Cholesterol	−0.006	0.003	0.02
HDL Cholesterol	0.006	0.003	0.03
LDL Cholesterol	0.006	0.003	0.04
Triglycerides	0.001	0.0005	0.014
Potassium	0.013	0.006	0.016
Interactions			
Magnesium→Total Cholesterol	0.008	0.003	0.011
Magnesium→HDL Cholesterol	−0.007	0.003	0.016
Magnesium→LDL Cholesterol	−0.007	0.003	0.03
Magnesium→Triglycerides	−0.0014	0.0005	0.01

Estimated parameters (beta), standard error (SE) and *p*-value shown for each significant variable considering interactions.

## Supplementary Materials

The following are available online at https://www.mdpi.com/2072-6643/12/9/2631/s1, Table S1: Bivariate correlation coefficients between cIMT and Magnesium levels with other variables in control volunteers.
