# Supplementary material for: Magnesium Levels Modify the Effect of Lipid Parameters on Carotid Intima Media Thickness
Source: Nutrients. 2020 Aug 28;12(9):2631. doi: 10.3390/nu12092631 (PMC7551902; doi:10.3390/nu12092631)
Supplement: Supplementary file 1 [file nutrients-12-02631-s001.pdf]

Supplemental Table S1. Bivariate correlation coefficients between cIMT and Magnesium levels with other variables in control volunteers

	cIMT		Magnesium	
Variable	r	p value	r	p value
Sex (Male)	0.260	<0.001	0.102	0.138
Race (Caucasian)	0.071	0.301	0.008	0.913
Age, years	0.623	<0.001	0.06	0.382
Smoker	0.040	0.561	0.014	0.844
Diabetes	-	-	-	-
Hypertension	0.324	<0.001	0.131	0.057
Dyslipidemia	0.199	0.004	0.063	0.364
BMI	0.248	<0.001	-0.055	0.427
Total Cholesterol	0.255	<0.001	0.023	0.740
HDL Cholesterol	0.006	0.934	-0.077	0.298
LDL Cholesterol	0.219	0.003	-0.001	0.990
Triglycerides	0.159	0.023	0.108	0.122
Magnesium	0.091	0.185	-	-
cIMT	-	-	0.091	0.185

Pearson´s correlation coefficients (r) and p-value are shown for each variable. Abbreviations: Body Mass Index (BMI), Carotid Intima-Media Thickness (cIMT).
